# Mechanisms of Phototoxic Effects of Cationic Porphyrins on Human Cells In Vitro

**DOI:** 10.3390/molecules28031090

**Published:** 2023-01-21

**Authors:** Yegor E. Yegorov, Khava S. Vishnyakova, Xiaowen Pan, Anton E. Egorov, Konstantin V. Popov, Liana L. Tevonyan, Galina V. Chashchina, Dmitry N. Kaluzhny

**Affiliations:** 1 Engelhardt Institute of Molecular Biology, Russian Academy of Sciences, 119991 Moscow, Russia; 2 Moscow Institute of Physics and Technology, Russian Academy of Sciences, 141701 Dolgoprudny, Russia; 3 Emanuel Institute of Biochemical Physics, Russian Academy of Science, 4 Kosygin Street, 119334 Moscow, Russia; 4 National Medical Research Center for Obstetrics, Gynecology and Perinatology Named after Academician V.I.Kulakov, 4 Oparina Street, 117997 Moscow, Russia

**Keywords:** porphyrins, porphyrin-Zn, human cells, necrosis, photodynamic therapy

## Abstract

The toxic effects of four cationic porphyrins on various human cells were studied in vitro. It was found that, under dark conditions, porphyrins are almost nontoxic, while, under the action of light, the toxic effect was observed starting from nanomolar concentrations. At a concentration of 100 nM, porphyrins caused inhibition of metabolism in the MTT test in normal and cancer cells. Furthermore, low concentrations of porphyrins inhibited colony formation. The toxic effect was nonlinear; with increasing concentrations of various porphyrins, up to about 1 μM, the effect reached a plateau. In addition to the MTT test, this was repeated in experiments examining cell permeability to trypan blue, as well as survival after 24 h. The first visible manifestation of the toxic action of porphyrins is blebbing and swelling of cells. Against the background of this process, permeability to porphyrins and trypan blue appears. Subsequently, most cells (even mitotic cells) freeze in this swollen state for a long time (24 and even 48 h), remaining attached. Cellular morphology is mostly preserved. Thus, it is clear that the cells undergo mainly necrotic death. The hypothesis proposed is that the concentration dependence of membrane damage indicates a limited number of porphyrin targets on the membrane. These targets may be any ion channels, which should be considered in photodynamic therapy.

## 1. Introduction

Porphyrins are the most common agents for photodynamic therapy. Various modifications of porphyrins allow them to be adapted to specific needs by selecting the molecular size, charge, spectral characteristics, etc. They were initially studied as DNA-binding porphyrins (TMPyP4), expanding their spectrum of activity over time. A number of studies have shown the possibility of antitumor, antiviral, and antimicrobial activity of the cationic porphyrin TMPyP4 [1,2,3,4].

The complex processes associated with the response of the immune system to photodynamic therapy suggest an immunomodulatory effect of porphyrins. Various forms of cell death combined with changes in gene expression and microbial response create the prerequisites for the study of porphyrins in connection with the therapy of noncancerous human pathologies [5].

The mechanism of action of porphyrins can be varied, including direct interaction with nucleic acids. Noncanonical structures, such as G-quadruplexes, formed by the telomeric ends of chromosomes are considered promising targets for cationic porphyrins. Such interaction with DNA suggests a directed action on telomeres, thereby inhibiting telomere elongation by telomerase. Metal ions coordinated in the core of the macrocycle modulate the interaction with DNA [6,7]. However, the introduction of zinc enhances the photochemical effect on DNA. It was previously shown that the introduction of zinc into tetracarboxymethyl-substituted pyridyl porphyrin leads to selective interaction and photooxidation of the telomeric sequence DNA [8,9].

Intensive research is still under way to find out how compounds with such biological potential can be used in cancer therapy, especially as they can induce biological effects in a telomere-independent manner. However, it has been shown that the target of cationic porphyrins can be not only telomeres, but also other targets, such as cell adhesion and migration. Using whole-exome sequencing methods, it was shown that, at low concentrations, TMPyP4 activates the expression of genes associated with cell migration [10]. TMPyP4-induced cell death by apoptosis in tumor cells is associated with altered gene expression and, therefore, TMPyP4 may represent a potential therapeutic option for cancer [11]. It is suggested that TMPyP4 can significantly contribute to a significant reduction in the spread of cancer cells and, consequently, a decrease in the survival of cancer cells. It is important to note that this effect may not be related to telomeres or telomerase [12].

Photoactivated DNA degradation in complex with porphyrins can serve as one of the possibilities for the directed action of porphyrins on certain DNA regions, the chemical modification of porphyrins enhances and activates such a “nuclease” action [13]. To explore this possible targeting, it is necessary to find out how the compounds are able to penetrate into the nucleus of the cell and, activated by light, cause damage to DNA.

All studies of porphyrins on cells can be roughly divided into “dark” and photodynamic. In studies without special illumination, changes in gene expression, suppression of telomerase activity, and a type of cell death are very well described. Such studies are characterized by long duration (days) and high concentration of porphyrins (tens of micromoles). For example, in [12,14,15,16], the TMPyP4 porphyrin at a concentration of 100 μM was used, and the incubation time was 48–96 h. Concentrations hundreds of times lower [17,18,19,20] were usually used in illumination experiments. For example, in Rapozzi et al. [20], the IC_50_ for TMPyP4 was 200 nM. We were more interested in studying the mechanisms of light-induced cell death.

**Figure 1:**
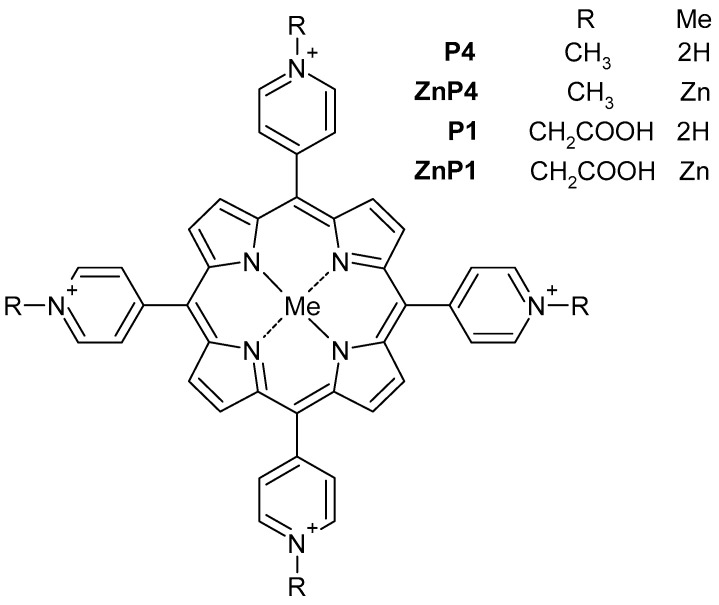
Schematics of the porphyrins used.

This work is a study of the photoinduced effect on tumor and “normal” cell lines by cationic porphyrins. We used two types of tetrapyridine porphyrins: cationic porphyrin P4 (TMPyP4) and its amphiphilic derivative porphyrin P1 containing carboxyl groups, as well as their zinc-containing analogues ZnP4 and ZnP1 (Figure 1). Primary culture of human mesenchymal stromal cells KO-16, an immortalized human embryonic fibroblast line (977-hTERT), and two cancer cell lines A549 and SK-N-SH were investigated. The results of the work showed a predominantly necrotic type of cell death under the action of porphyrins + light. The work revealed a number of methodological difficulties that are discussed and will be useful for researchers.

## 2. Materials and Methods

### 2.1. Compounds, Lighting

The derivatives of tetrapyridylporphyrins studied in the work are shown in Figure 1. The P4 was TMPyP4 (Sigma Aldrich, Saint Louis, MO, USA). The compounds ZnP4, P1, and ZnP1 were obtained as described previously [8,21,22]. Stock solutions with a porphyrin concentration of 10 mM in DMSO (PanEko, Moscow, Russia) were used. The cells were illuminated using blue light (465 ± 15 nm) of Kvant-C transilluminator (Helicon Co., Moscow, Russia). Radiation power was 4 mW/cm^2^.

### 2.2. Cells

The following cell cultures were used in this work: 977hTERT—human embryonic fibroblasts expressing the protein component of telomerase [23]; SK-N-SH—a culture of human neuroblastoma; A549—an adenocarcinoma cell line derived from human alveolar basal epithelial cells, kindly provided by Dr. Maria Kost-Alimova (Karolinska Institute, Solna, Sweden); KO-16—a primary culture of human mesenchymal stromal cells, kindly provided by A.A. Doktorov (NITSBMT VILAR, Moscow, Russia). 

Cells were cultured at 37 °C in an atmosphere of 5% carbon dioxide in DMEM (PanEko, Moscow, Russia) with a glucose content of 4.5 g/L and the addition of glutamine, gentamicin (40 μg/mL), and 10% embryonic bovine serum (Biolot, Saint Petersburg, Russia). Trypsin solution (PanEco, Moscow, Russia) was used for cell reseeding.

### 2.3. MTT Assay

Cell sensitivity to cationic porphyrins P1, P4, ZnP1, and ZnP4 was tested in a standard spectrophotometric (MTT) assay. Cells were grown for 24 h in 96-well multiplates, in 190 µL of culture medium. Subsequently, the test substances were added to each well. Each concentration was tested three times. To examine the cytotoxicity of porphyrins, two parallel experiments were performed in different conditions (light/lack of light). In the absence of light, cells were incubated with compounds for 1 and 24 h. In the presence of light, cells were incubated with porphyrins for 1 h, and then irradiated with blue light for 30 min and left at 37 °C for 1 and 24 h.

Next, 20 µL of MTT solution (0.5 mg/mL) was added to plates, and cells were incubated for 2 h. The medium was aspirated, and formazan crystals were dissolved in 100 µL of dimethyl sulfoxide. Absorbance was measured at 550 nm on a plate reader (Tecan Spark, Tecan, Männedorf, Switzerland). Three independent measurements were averaged. The same sample of cells without porphyrins was used as a control. 

### 2.4. Colony Formation Assays

Colony formation assays were performed as described in [24]. Briefly, the cells were counted in a Goryaev chamber, and 200 cells were seeded into 10 cm culture dishes (Costar, Coppell, TX, USA) and grown in DMEM medium (PanEco, Moscow, Russia) supplemented with 15% FBS (HyClone, Logan, UT, USA) in the presence of 5% CO_2_. On day 7, the cells were fixed with 70% ethanol and stained with 0.1% methylene blue. Cell staining was followed by quantification of colonies. We defined a colony to be of any size, including those consisting of only one cell. Each culture dish was placed on a grid; cell images were analyzed under a stereomicroscope. Positions of colonies and cell numbers in individual colonies were marked on the grid. The cell count in a colony was rounded up to the nearest 2^n^ number. For example, if the number of cells in a colony was 3 or 4, it was recorded as 4; if it was from 5 to 8, was recorded as 8; if it was from 129 to 256, was recorded as 256. 

Since the number of colonies in each dish differed from each other, we used a value of N% for comparison. The number of colonies N of the same size was summed up, and the value was divided by the total number of colonies analyzed. We used three Petri dishes for each experimental setting during the experiments. Each experiment was repeated three times. Counting was performed independently by two people (K.S.V. and X.P.). Thus, the graphs of the distribution of colonies by size reflect the average of the nine dishes, counted by two independent persons.

### 2.5. Trypan Blue Test

Part of the medium was removed from the Petri dishes with growing cells, leaving 0.5 mL, to which 0.5 mL of 0.4% trypan blue solution (Gibco, Waltham, MA, USA) was added. After 2 min, the observation was started.

### 2.6. Microscopy

Cell morphology was determined using a Diaphot inverted phase-contrast microscope (Nikon, Tokyo, Japan) and a Nikon D5000 camera. Fluorescent images were photographed using an Olympus BX53 microscope.

### 2.7. Spectroscopy

Electronic absorption spectra were obtained using a Shimadzu UV-3101PC spectrophotometer in the range of 350–800 nm in a quartz cuvette (optical path length 1 cm). Molar extinction coefficients were used from [25]. 

## 3. Results

### 3.1. Effect of Porphyrins on Colony Formation

**Figure 2:**
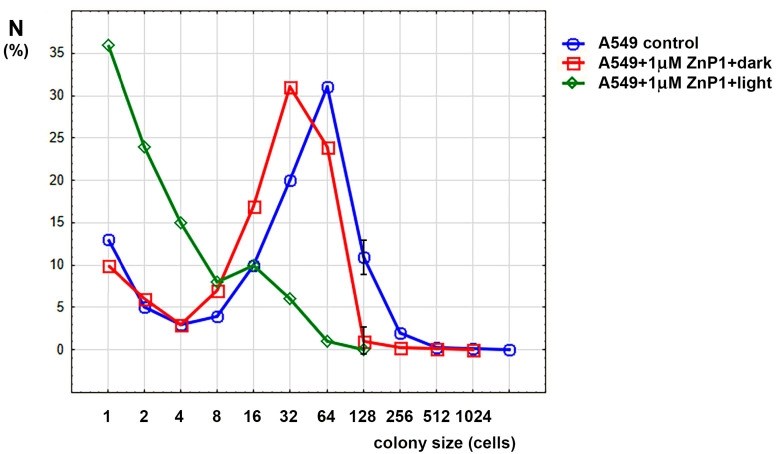
Colony size distribution in experiments with A549 cells. Only significant differences between control and dark porphyrin (mean ± SD) are shown.

The colony formation assay is a comprehensive test for cell viability including various aspects such as cell death and cell exit from the cell cycle due to damage and is the most sensitive. This test showed that, even without special illumination, porphyrins have a certain dark toxicity (Figure 2).

One can see from the Figure 2 that dark treatment with porphyrin slightly shifted the graph to the left, halving the modulus of distribution. When exposed to light (5 min), the distribution peak shifted further to the left, and the fraction of nondividing cells (single-cell colonies) also increased. Changes in the maximum size of the colonies are shown in Table 1.

Thus, it is shown that porphyrins can inhibit cell division and colony growth, which led to the idea of studying the mechanisms of their action on cells.

### 3.2. Dependence of Porphyrin Toxicity on Concentration and Time after Light Irradiation (MTT Test)

On three cell lines, we examined the cytotoxicity of cationic porphyrins P1, P4, and their modifications ZnP4 and ZnP1 with zinc in the porphyrin macrocycle. 

**Figure 3:**
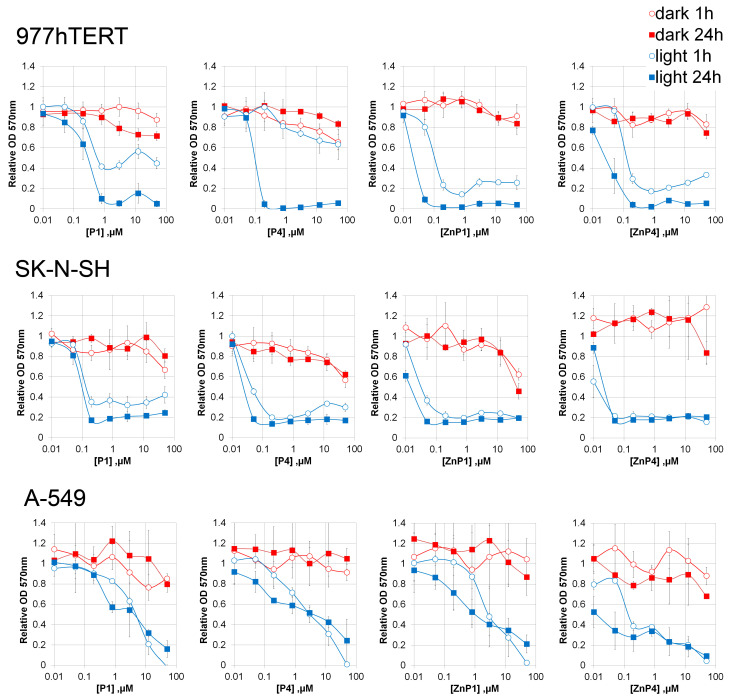
Effect of porphyrins and illumination on the metabolic activity of cells. The duration of illumination was 30 min. The optical density of the treated samples was related to the untreated ones. Results of three independent experiments were averaged; the mean ± SD is shown.

In the absence of light, the cationic porphyrins revealed low cytotoxicity (Figure 3), manifested at high concentrations (>50 μM). The maximum effect of cytotoxicity was revealed on the cells line of immortalized fibroblasts (977hTERT).

In the presence of blue light, there was a significant decrease in the viability of all cell lines (concentrations 50 nM). It took 1 h after light irradiation to stop the mitochondrial metabolism of cells. The lowest cytotoxicity was manifested on the A-549 cell line (human lung carcinoma). Nevertheless, micromolar concentrations of porphyrins caused cell death during 30 min of light irradiation. The most cytotoxic was porphyrin ZnP4.

An unexpected result of the experiments with MTT was that the toxic effect reached a plateau (with a nonzero level) at high concentrations of porphyrins for all the cells studied.

### 3.3. Study of Cellular Permeability with Trypan Blue

The trypan blue test shows the permeability of cell membranes to the dye and indicates the loss of membrane barrier function, which is usually a sign of necrotic cell death. If the dye enters the cell, the membrane is also permeable to ions and water.

**Figure 4:**
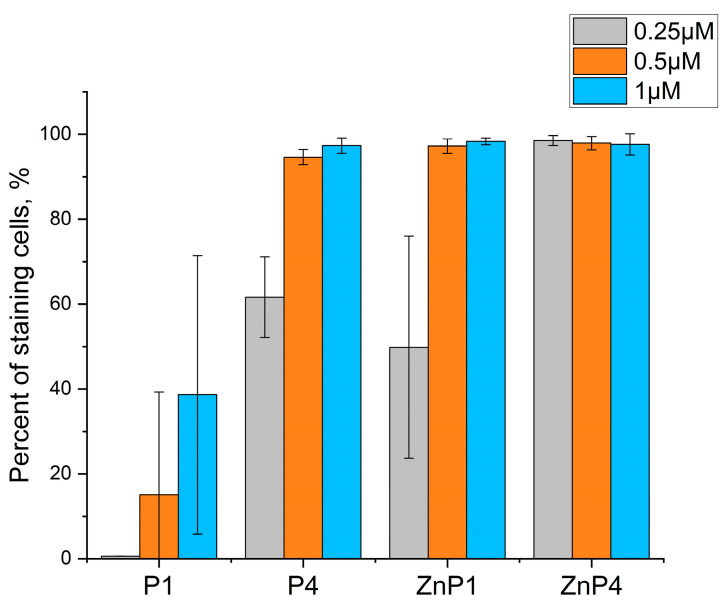
Penetration of trypan blue into SK-N-SH 1 h after illumination. Bars represent the mean ± SD of three independent experiments.

**Figure 5:**
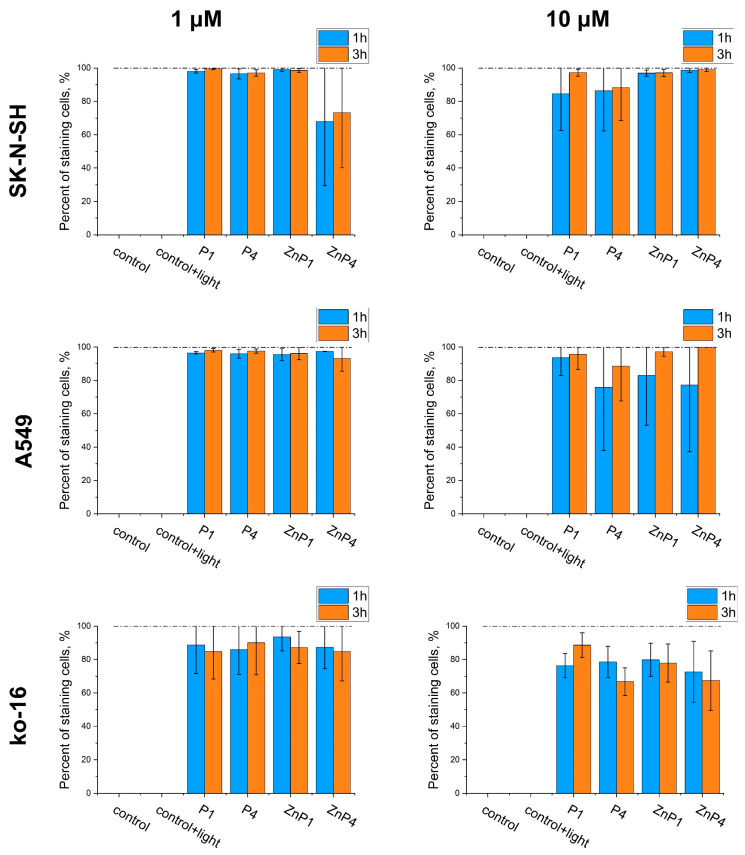
Comparison of the toxic effects of porphyrins in high concentrations by trypan blue staining. Counts were performed 1 and 3 h after illumination. Bars represent the mean ± SD of three independent experiments.

These experiments were performed under the same conditions as the MTT test and gave similar results. At relatively low concentrations of porphyrins, an increase in the proportion of stained cells with increasing concentration was observed (Figure 4). Porphyrins at concentrations of 1 and 10 μM yielded similar results (Figure 5).

Thus, experiments with trypan blue confirmed the results of the MTT test that the toxic effect plateaus. The toxicity of porphyrins changed little when the concentration was increased (Figure 5).

### 3.4. Morphological Studies of Porphyrins Action

**Figure 6:**
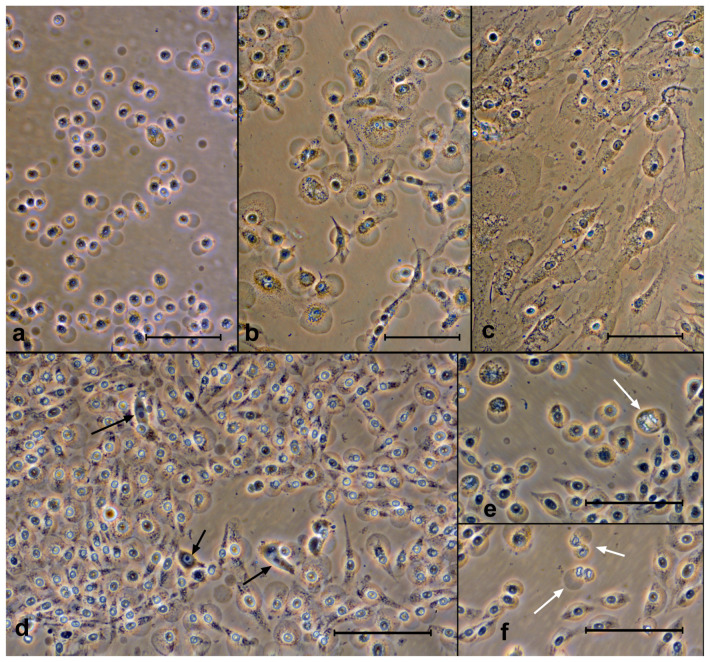
Cells after exposure to porphyrins with light. The swollen cells can be seen. Top row—after 3 h: (**a**) SK-N-SH (ZnP4, 10 μM), (**b**) A549 (ZnP4, 10 μM), and (**c**) Ko-16 (P1, 10 μM). Bottom row—SK-N-SH cells after 24 and 48 h after exposure: (**d**) P1, 1 μM, (**e**) ZnP4, 10 μM, and (**f**) ZnP1, 10 μM. The vast majority of cells are attached, and the blebs are preserved. Black arrows indicate live cells, while white arrows indicate conserved mitoses. Bars = 100 μM. Phase contrast, digital contrast.

The main morphological signs in cells after the action of porphyrins are blebs (Figure 6). They appear rather quickly (mostly during the first hour, after which their proportion practically does not increase). They do not disappear even after 24 or 48 h. The porphyrins differed in the effectiveness of “blebogenesis”; ZnP4 had the strongest effect, followed by ZnP1, P4, and P1. This sequence repeated the data of the MTT test and the experiments with trypan blue.

In experiments with different concentrations of porphyrins (1 and 10 μM), we observed only single live cells after 24 h (Figure 6d). It was not possible to visually determine the differences between the 1 μM and 10 μM experiments.

### 3.5. Porphyrin Penetration into the Cell

**Figure 7:**
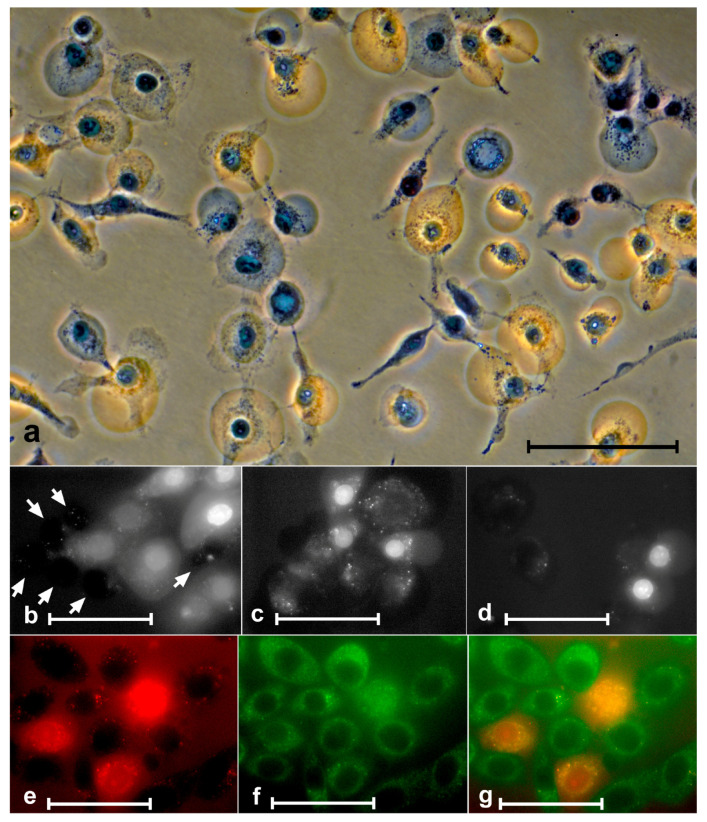
Penetration of trypan blue and porphyrins into cells. (**a**) A549 cells after treatment with 10 µM ZnP1 + light stained with trypan blue. Some of the blebs were stained with trypan blue and some were not. The middle row SK-N-SH cells after the action of 1 µM ZnP1 + light. (**b**) Six unstained porphyrin cells (white arrows) and eight stained cells (with different intensity). The stained cells are significantly larger in size (they are swollen). (**d**) Two stained and two unstained cells of about the same size. The bottom row—staining of the nucleus and cytoplasm of SK-N-SH cells (1 µM ZnP1 + light) occurred synchronously: (**e**) porphyrin, (**f**) autofluorescence, and (**g**) merged images. Bars = (**a**) 100 µM, and (**b**–**g**) 50 µM; (**a**) phase contrast, digital contrast.

Without light irradiation, the porphyrins under study did not stain cells. We were able to observe only isolated cases of staining of damaged cells. The appearance of blebs and swelling of cells is usually evidence of membrane damage and is associated with an increase in its permeability. In experiments with trypan blue, we were convinced of this (Figure 7a).

It is clear from the figure that, indeed, at some stage after the irradiation, the plasmalemma permeability increased and reached the stage when trypan blue entered the cell. Similar to trypan blue, porphyrins, at some point after exposure to porphyrins + light, started to pass into cells (Figure 7b–g). Penetration of porphyrins into cells was usually preceded by swelling.

Staining of cytoplasm and nuclei occurred synchronously. In cells with stained cytoplasm, we always observed staining in the nucleus (Figure 7e–g).

### 3.6. Trypsinization Procedure

**Figure 8:**
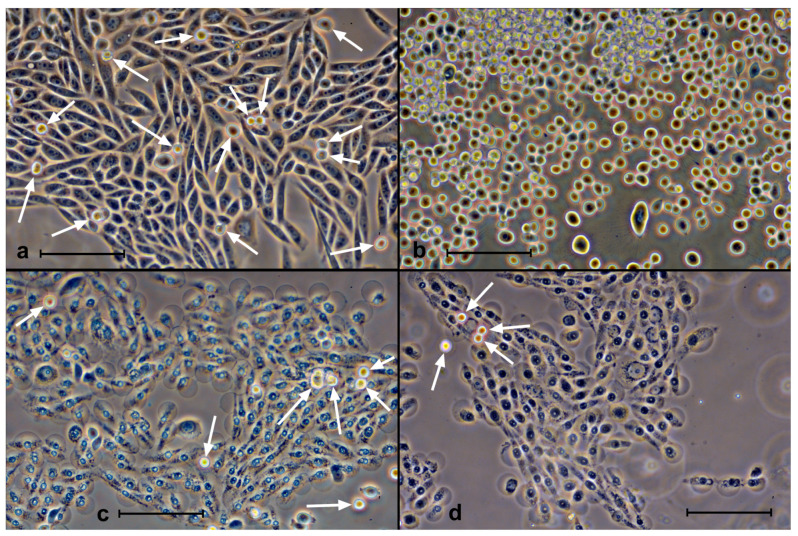
Effects of trypsin on SK-N-SH cells 2 h after incubation with ZnP4 (10 μM) and illumination (10 min). (**a**) Initial culture after incubation with porphyrin; (**b**) 5 min after trypsin action, with almost all cells in suspension, indistinguishable from the action on cells without porphyrin; (**c**) culture after incubation with porphyrin and illumination, with most cells showing signs of fatal membrane damage; (**d**) 5 min exposure to trypsin on cells irradiated with porphyrin, with no visible response to trypsin. White arrows in (**a**,**c,d**) indicate suspended cells. Phase contrast, digital contrast. Bars = 100 μm.

In order to remove the cells from the substrate (transfer the attached cells into suspension), they are treated with a trypsin solution. The procedure is standard, suitable for almost all cell types. We found that this process stopped working in the case of cells with porphyrin irradiated with light (Figure 8). Trypsin-treated cells do not change their morphology and do not detach from the substrate. Very few rounded cells are found in the suspension. It is possible that some of them were in suspension even before trypsin treatment (mitotic and dying cells). The observation of such “trypsin resistance” was true for all three cell types (KO-16, A549, and SK-N-SH). In the presence of porphyrins, but without illumination, trypsin worked “correctly”, i.e., the cells were rounded up and detached.

### 3.7. Annexin V Staining

We were interested to find out to what extent apoptotic cells were represented in our experiments. It is known that one of the signs of apoptosis is the appearance of phosphatidylserine in the outer plasmalemma leaflet, which moves there from the inner leaflet. Because we found that cells do not detach from the substrate by trypsin after treatment with porphyrins and light, it was impossible to apply the standard flow measurement technique. Therefore, cells were stained with an annexin–FITC conjugate (FITC Annexin V, BD Pharmingen, no. 556420) recognizing phosphatidylserines, and microscopic observations were performed.

**Figure 9:**
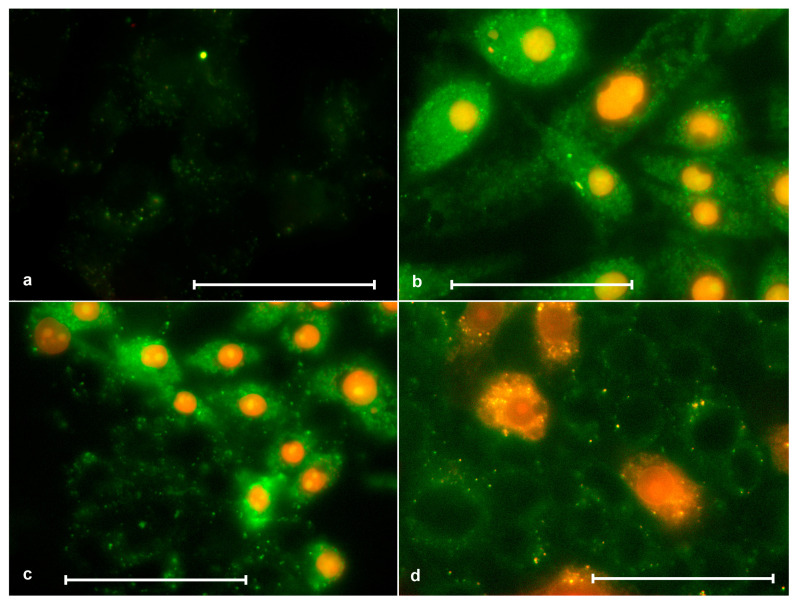
Staining of SK-N-SH cells with annexin (green color) and porphyrins(red color). (**a**) Control without porphyrin, where a faintly stained cytoplasm can be seen; (**b**) after 2 h of action of ZnP4 (0, 25 μM) + light 10 min, where all nuclei were stained with porphyrin, and different cells were stained with annexin to different degrees, but brighter than the control; (**c**) after 2 h of action of P4 (0.25 µM) + 10 min light, where some cells are weakly stained with annexin and not stained with porphyrin (normal), while some are strongly stained with annexin and stained with porphyrin (necrosis); (**d**) similar to (**c**), but using P1 (1 μM). Bars = 50 μM.

The observations showed that, among the attached cells, we could see only normal cells (weakly stained with annexin and not stained with porphyrin) or necrotic cells (well stained with annexin and stained with porphyrin) (Figure 9). 

### 3.8. Aggregation Studies of Porphyrins

It has been suggested that, when the concentration increases, porphyrins begin to aggregate with each other, which leads to a decrease in the production of reactive oxygen species, which can reduce the toxic effects.

**Figure 10:**
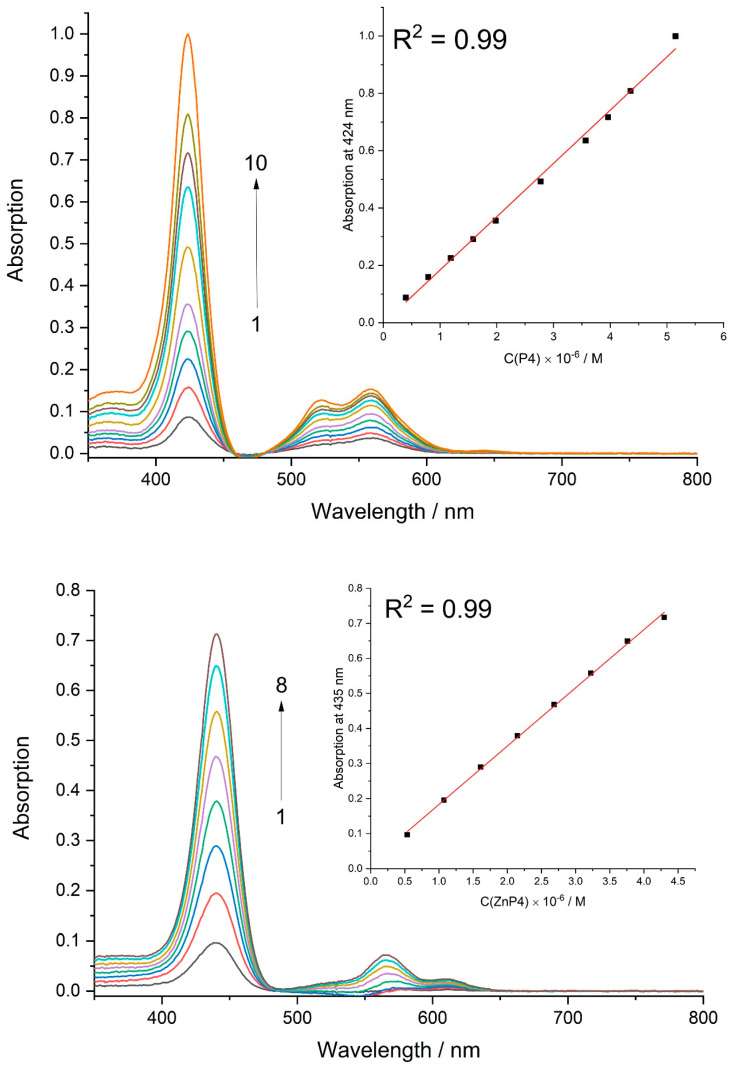
Absorption spectra of P4 at different concentrations ([P4] × 10^6^ M: 1 − 0.4, 2 – 0.8, 3 – 1.2, 4 – 1.6, 5 – 2.0, 6 – 2.8, 7 – 3.6, 8 – 4.0, 9 – 4.4, 10 – 5.1) and ZnP4 at different concentrations ([ZnP4] × 10^6^ M: 1 − 0.5, 2 − 1.1, 3 − 1.6, 4 – 2.1, 5 – 2.7, 6 – 3.2, 7 – 3.7, 8 – 4.3) in culture media.

Aggregation studies of porphyrins in DMEM media with all supplements were performed using UV/Vis spectroscopy. Upon increasing the concentration of ZnP4 and P4 up to around 5 × 10^−6^ M, no deviations in spectral shape were observed (Figure 10). The linear dependencies between optical absorption at 435 nm (for ZnP4) and at 424 nm (for P4) and the concentration of corresponding porphyrin were obtained. 

Thus, the hypothesis of porphyrin aggregation in solution was rejected.

## 4. Discussion 

Experiments showed the strongest dependence of the toxic effect of porphyrins on light. It is the residual effect that apparently explains the slight dark toxicity in the experiments on colony formation and MTT, since the work took place under normal lighting conditions.

We encountered a number of methodological difficulties in our work. Experiments on cell staining with porphyrins were poorly reproducible. This was due to two factors: mechanical instability of swollen cells and the observation procedure itself. The swollen cells could break (rupture) simply because of a change of medium, greatly affecting the result. To see the fluorescence of porphyrin, it should be illuminated with light that gives a photodynamic effect. Keep in mind that, in fluorescence microscopy, the excitation flux is extremely intense and can have a significant effect on cell viability in a few minutes. In other words, the result literally changes before our eyes.

In the process, it was found that treatment of the cells with porphyrins and light resulted in trypsin resistance. The cells could not be suspended and, accordingly, it was impossible to make an assay in flow. The cell yield was a few percent. In the end, it became clear that transferring the cells into suspension implied that the cells were alive. Normally, when trypsin acts, the cells round off, retracting their processes. The cytoskeleton is working, and all these processes require energy supply, which perforated cells cannot have. Many have advised us to use a flow assay to find out the types of cell death. As we see now, this analysis would lead to erroneous conclusions, because, in the process of sample preparation, there would be a powerful selection against necrotic cells, which, in our samples after the action of porphyrins and light, are the overwhelming majority.

We consider the most interesting result to be the nonlinear dependence of the toxic effect on the concentration of porphyrins. The first hypothesis that arose to explain the dependence obtained is that porphyrins can interact with each other to form aggregates. Such cases have been described in the literature [26,27,28,29]. 

In this case, one would expect that the dimers (or aggregates in general) change their quantum yield and form fewer reactive oxygen species. Therefore, toxicity is reduced. In addition, the aggregates have a higher molecular weight, which makes it more difficult for them to penetrate into the cells. However, direct measurements showed that, under culture medium conditions, the spectra of porphyrins do not change with increasing concentration. Another possibility remains that porphyrins form aggregates directly on the membrane, and such variants have been described in the literature [30]. Considering that our studied porphyrins have rather weak hydrophobicity [21], we consider it unrealistic.

There is another hypothesis that there are rapidly inducible pumps in cells pumping out xenobiotics, including porphyrins [31,32]. However, the results of the toxicity experiments were not influenced by the time of dark preincubation with porphyrins within 1 h (data not shown). In addition, since we did not observe penetration of porphyrins into living cells, such a hypothesis contained a contradiction in itself and was rejected. 

Our third hypothesis is that porphyrins react with a limited number of targets on the cell surface. Given the weak hydrophobicity of the porphyrins used [21], we can assume that interaction with protein targets on the membrane is crucial. One can even assume that the targets are formed during the interaction, as described in [33,34,35].

It is surprising that, in our experiments with 1 μM and with 10 μM porphyrins under strong light (30 min), after 24 h, in both cases, we observed only individual living cells. Morphological observations indicated that, under the action of porphyrins and light, the membrane permeability to small molecules (e.g., ions and water) increases first, and only then does the permeability to porphyrins appear. The photographs showed that the porphyrin-stained cells are somewhat larger (swollen) (Figure 7b–g). Porphyrins only enter already swollen cells. It can be assumed that there are different targets on the membrane that allow molecules of different sizes to pass through. The same applies to permeability to trypan blue. Ambiguities in the interpretation of the photographs may arise due to the essential fragility of the membrane of swollen cells. Exposure to a medium change procedure can cause these cells to rupture.

Most cells in our experiments died necrotically; the photos of cells 24 h after exposure are clear proof of this. Morphologically, the cells were quite similar to live cells, no changes in the shape of the nucleus occurred, nuclei were visible, and cells retained their initial spreading state and did not lose contact with the substrate. Evidence of their death was as follows: blebs, high contrast of cytoplasm (due to withdrawal of low-molecular-weight components while retaining high-molecular-weight ones), and free space frequently visible around nuclei.

It can be assumed that some cells (a small part) are detached from the substrate and undergo apoptosis as a result of the action of porphyrin and light. We did not investigate this question. In any case, these cells are a minority. Among the attached cells, we could not find cells corresponding to apoptosis by annexin and porphyrin staining using microscopy (Figure 9). Probably, the yield of apoptotic cells can be higher when porphyrins act weakly (low concentration and weak illumination).

If the hypothesis of a limited number of targets is correct, then it is possible to have some artificial effect on the targets that would modulate the overall result. The type of cell death in photodynamic cancer therapy can be double-edged. With predominantly apoptotic death, patients would tolerate the therapy more easily; with predominantly necrotic death, severe inflammation would develop. This inflammation would attract cells of the immune system and possibly enhance its cytotoxic action to destroy the tumor.

## Figures and Tables

**Table 1:** Maximum colony sizes in experiments with porphyrin and light.

Cells	Control	0.1 μM ZnP1	1 μM ZnP1	0.1 μM ZnP1+ 5 Min Light	1 μM ZnP1+ 5 Min Light
A549	1024	1024	512	512	64
977hTERT	64	32	32	16	8

## Sample Availability

Not available.
